# Hot Deformation Behavior Considering Strain Effects and Recrystallization Mechanism of an Al-Zn-Mg-Cu Alloy

**DOI:** 10.3390/ma13071743

**Published:** 2020-04-09

**Authors:** Lei Luo, Zhiyi Liu, Song Bai, Juangang Zhao, Diping Zeng, Jian Wang, Jing Cao, Yangcheng Hu

**Affiliations:** 1 Light Alloy Research Institute, Central South University, Changsha 410083, China; kevinluo03219@163.com; 2 Key Laboratory of Nonferrous Metal Materials Science and Engineering, Ministry of Education, Central South University, Changsha 410083, China; juangangzhao@163.com (J.Z.); zengdiping001@163.com (D.Z.); wangjian1212@126.com (J.W.); jingcao@csu.edu.cn (J.C.); csuhuyangcheng@163.com (Y.H.); 3 School of Material Science and Engineering, Central South University, Changsha 410083, China; 4 National Key Laboratory of Science and Technology for National Defense on High-Strength Structural Materials, Central South University, Changsha 410083, China

**Keywords:** Al-Zn-Mg-Cu alloy, constitutive model, processing map, microstructural evolution, dynamic recrystallization

## Abstract

The hot deformation behavior of an Al-Zn-Mg-Cu alloy was investigated by hot compression test at deformation temperatures varying from 320 to 440 °C with strain rates ranging from 0.01 to 10 s^−1^. The results show that the Mg(Zn, Cu)_2_ particles as a result of the sufficient static precipitation prior to hot compression have an influence on flow softening. A constitutive model compensated with strain was developed from the experimental results, and it proved to be accurate for predicting the hot deformation behavior. Processing maps at various strains were established. The microstructural evolution demonstrates that the dominant dynamic softening mechanism stems from dynamic recovery (DRV) and partial dynamic recrystallization (DRX). The recrystallization mechanism is continuous dynamic recrystallization (CDRX). The microstructure observations are in good agreement with the results of processing maps. On account of the processing map and microstructural observation, the optimal hot processing parameters at a strain of 0.6 are at deformation temperature range of 390–440 °C and strain rate range of 0.010–0.316 s^−1^ with a peak efficiency of 0.390.

## 1. Introduction

Al-Zn-Mg-Cu alloys are extensively used for structural applications of aerospace, petroleum and gas, and automotive industries (aircraft fuselage, drill pipe, etc.) due to their high strength-to-weight ratio, good stress corrosion cracking resistance, and high fracture toughness [1,2,3,4,5]. Meanwhile, hot forming (rolling, forging, extrusion, etc.) is a significant processing technique for manufacturing these structural components. As a newly developed Al-Zn-Mg-Cu alloy used in oil drilling pipe, the studied alloy shows a relatively high strength and excellent thermal stability as compared to the conventional Al-Zn-Mg-Cu alloy with low Zn/Mg ratio. For the former alloy, high Zn/Mg ratio leads to a denser precipitation of η′ phase. Generally, oil drilling pipes are fabricated by hot extrusion. The performance of materials mainly depends on the final microstructure, which, in turn, is affected by the forming parameters. However, material flow behavior is often very complicated during hot extrusion. Various metallurgical phenomena often take place, which greatly influence the material flow behavior and final microstructure [6,7,8]. Therefore, an investigation of the hot deformation behavior, microstructural evolution, and corresponding mechanism of the studied alloy is quite crucial for industrial production, as the industrial manufacturing processes can benefit from the research results.

The flow behavior of alloys under different deformation conditions is usually described by the constitutive model. Many efforts have been performed to derive several suitable constitutive models to characterize the hot deformation behavior of aluminum alloys, such as 2026 [9], 7085 [10], and 7150 aluminum alloy [11]. However, the previous studies seldom concern the effect of strain, which possesses a significant influence on accurate prediction of the flow behavior of materials. Recently, Zhang et al. described the deformation behavior of Cu-Zr-Ce alloy and proposed a revised Arrhenius-type constitutive model by considering the compensation of strain. It was found that the developed constitutive equation exhibited an excellent prediction capability [12]. A similar method was employed by Cai et al. to develop two Arrhenius-type constitutive models with and without the strain compensation for AZ41M magnesium alloy. Their results stated that the revised model correlated well with the experimental results and accurately predicted the flow behavior of AZ41M [13]. Additionally, a revised Arrhenius-type constitutive model was used for predicting the flow behavior in Ti60 titanium alloy [14].

The grain structure varies greatly by different processing parameters, which leads to different recrystallization mechanisms. There are two main dynamic recrystallization (DRX) mechanisms (i.e., continuous dynamic recrystallization (CDRX) and discontinuous dynamic recrystallization (DDRX)). CDRX is characterized by progressive subgrain rotation, whereas DDRX is represented by nucleation and growth. Evolution of grain structures during hot deformation has attracted considerable interest in many investigations concerning recrystallization mechanism. In recent years, Luo et al. found numerous newly formed subgrains in the grain interiors as 7A09 aluminum alloy deformed at low temperatures (360–460 °C) with high strain rates (0.01–10 s^−1^), which was considered to be the result of CDRX [15]. The results obtained by Wang et al. showed that many recrystallized grains were found at grain boundaries as Al-Cu-Li alloy deformed at temperatures of 500 and 550 °C with two strain rates of 0.1 and 0.01 s^−1^, which were caused by DDRX [16]. Additionally, similar investigation was carried out for nickel-based superalloy [17].

Recently, many studies on Al-Zn-Mg-Cu alloy have been performed. Lin et al. found that the higher fatigue crack propagation (FCP) resistance of Al-Zn-Mg-Cu alloy was detected in T7351 condition rather than T7651 condition [18]. Cheng et al. proposed that the excellent mechanical properties of the nanocrystalline Al-Zn-Mg-Cu alloy resulted from the precipitated second-phase particles [19]. Furthermore, several investigations on the flow behavior and microstructure of Al-Zn-Mg-Cu alloy were performed. Shi et al. proposed a decline ratio map to study the hot deformation behavior of Al-Zn-Mg-Cu alloy, and they pointed out that the flow behavior was sensitive to the deformation temperature and strain rate [20]. Wang et al. developed the processing map and the constitutive model without considering the effect of strain for Al-Zn-Mg-Cu alloy and obtained the optimum parameters [21]. Zhao et al. investigated the hot deformation behavior and recrystallization behavior of Al-Zn-Mg-Cu alloy by considering the compensation of strain [22]. Zang et al. discussed the effect of initial microstructure on the hot deformation behavior of Al-Zn-Mg-Cu alloy. However, strain compensation was not considered in developing the constitutive model [23]. Despite the progress achieved in the abovementioned studies in exploring the flow behavior and recrystallization mechanism of Al-Zn-Mg-Cu alloys, few studies have focused on the hot deformation behavior considering strain effects and discussed the relationship between the second phase and the microstructural evolution in detail.

In present work, the hot compression test of the sample is performed under different deformation conditions. The constitutive model compensated with strain according to the Arrhenius-type model is developed and validated by experimental results. Then, the processing maps at various strains are established. In addition, the microstructural evolution and recrystallization mechanism of different deformation conditions are analyzed to confirm the processing map. The effect of the second phase on microstructural evolution is also discussed in detail. Finally, the optimal hot processing parameters of Al-Zn-Mg-Cu alloy are obtained.

## 2. Materials and Methods

**Figure 1:**
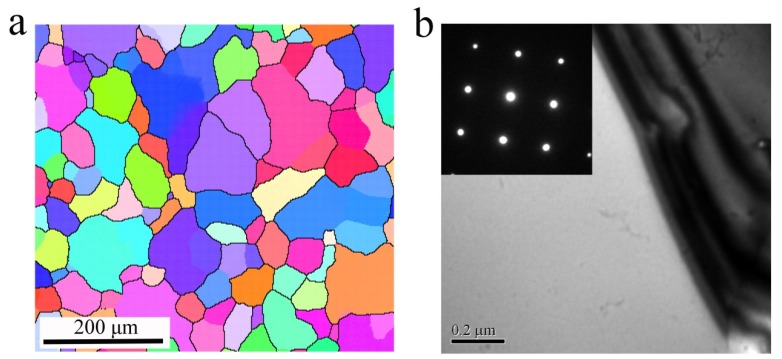
Microstructures of the homogenized Al-Zn-Mg-Cu alloy: (**a**) EBSD and (**b**) TEM images.

In this work, the chemical composition of the studied Al-Zn-Mg-Cu alloy is listed in Table 1. The cast ingot was subjected to a two-step homogenization that consisted of a low-temperature step at 420 °C for 24 h followed by a high-temperature step at 465 °C for 72 h, after which it was machined to cylindrical compressive samples that were 10 mm in diameter and 15 mm in height. The initial microstructure of homogenized samples with a mean grain size approximately 60 μm is shown in Figure 1a. Figure 1b shows that few precipitates appear in the homogenized samples. The hot compression tests were performed at temperatures of 320, 350, 380, 410 and 440 °C with strain rates of 0.01, 0.1, 1 and 10 s^−1^ on a Gleeble-3500 testing system (Dynamic Systems Inc., New York, NY, USA). A graphite lubricant was utilized between the samples and the press indenters to maximize lubrication during the tests. Prior to loading, the samples were heated to the target temperature with 2 °C/s heating rate and then held for 3 min. The samples were compressed to 60% reduction and immediately water quenched.

The microstructures of samples were investigated at the center of the axial section by TEM and EBSD. The TEM foils were mechanically ground to 100 µm, punched subsequently into 3 mm discs, and then twin-jet electro-polished in a 20% HNO_3_ and 80% CH_3_OH solution with the temperature at about −25 °C. The samples for EBSD were prepared using the same method as those for TEM preparation, except that the twin-jet electro-polishing time was shorter than that of TEM samples. The observation of TEM samples were performed on a FEI Tecnai-G2 20 electron microscope (FEI Inc., Hillsboro, OR, USA) at 200 kV. The EBSD tests were operated on the ZEISS EVOMA instrument (ZEISS Inc., Oberkochen, Germany) equipped with an Oxford EBSD attachment (Oxford Instruments Inc., Abingdon, UK). TSL OIM Analysis 5 software (EDAX Inc., Mahwah, NJ, USA) was used for analyzing EBSD data.

## 3. Results and Discussion

### 3.1. Flow Stress Behavior

**Figure 2:**
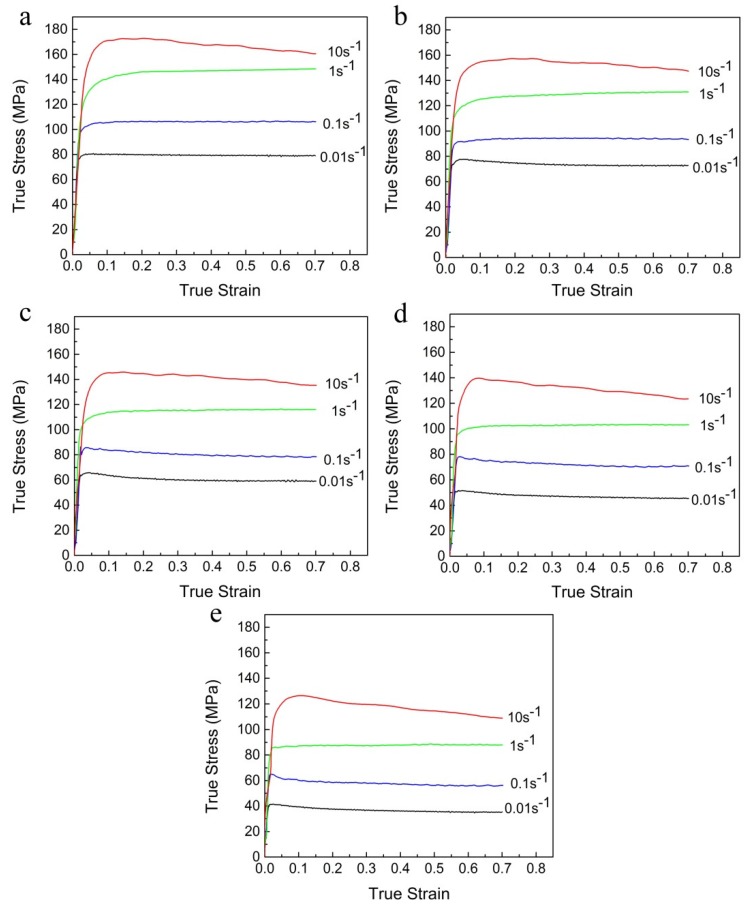
True stress–strain curves at deformation temperatures of (**a**) 320 °C, (**b**) 350 °C, (**c**) 380 °C, (**d**) 410 °C, and (**e**) 440 °C.

The experimental true stress–strain curves of Al-Zn-Mg-Cu alloy compressed under different deformation conditions are presented in Figure 2. It can be clearly observed that all the true stress–strain curves demonstrate a similar essential feature. The true stress–strain curves present initial work hardening (WH) followed by dynamic softening (DS) or inflexible state behavior. At the initial stage of deformation, the dislocation continually increases and multiplies rapidly. In addition, the formation of sufficient static precipitates occurs during the sample preheating and stabilization at the compression temperature [24,25]. Thus, the true stress dramatically increases, and the WH is dominant. At the softening stage of deformation, the density of accumulated dislocations exceeds a critical value, and the dislocation movement is sufficiently driven by the gradually accumulated energy. Moreover, severe deformation heating can also be generated as the hot compression tests are performed at high strain rates [26,27]. Consequently, the true stress decreases, and DS occurs at this stage. At the steady stage of deformation, the true stress keeps an inflexible state, owing to a dynamic equilibrium between the WH and DS.

Meanwhile, Figure 2 shows that the peak flow stress decreases with the increasing deformation temperature or the decreasing strain rate. It should be associated with the fact that the increasing in deformation temperature can provide higher grain boundaries mobility for the nucleation and growth of DRX grains, and the decreasing in strain rate can obtain longer time for the movement of dislocations and the accumulation of deformation energy. Additionally, the thermal activation of atoms intensified with the increase of deformation temperature leads to further movement of dislocation and vacancy [28]. Thus, the dynamic recovery (DRV) and DRX caused by the slip and climb of dislocations are improved. In addition, with the increasing deformation temperatures, more and more potential second phases tend to continue dynamic coarsening or dissolution during hot compression, which lowers the alloy strength [10]. Therefore, the DS behavior is improved, causing a decrease in the peak flow stress.

### 3.2. Constitutive Model for Flow Stress

The Arrhenius-type model is widely used to represent hot deformation behavior [29], as shown in Equation (1).

(1) $$\dot{\varepsilon }=\{\begin{array}{ll} A_{1}\sigma^{n_{1}}exp\left(-\frac{Q}{RT}\right) & \left(\alpha \sigma <0.8\right)\\ A_{2}exp\left(\beta \sigma \right)exp\left(-\frac{Q}{RT}\right) & \left(\alpha \sigma >1.2\right)\\ A{\left[sinh\left(\alpha \sigma \right)\right]}^{n}exp\left(-\frac{Q}{RT}\right) & \left(\mathrm{for}\;\mathrm{all}\;\sigma \right) \end{array}$$

where $\dot{\varepsilon }$ represents the strain rate (s^−1^); *A_1_*, *A_2_*, *A*, *n_1_*, *n*, *α*, and *β* are material constants, and *α* = *β*/*n_1_*; *Q* means the deformable activation energy (kJ mol^−1^); *R* represents the gas constant (8.314 J mol^−1^ K^−1^); *T* is the absolute temperature (K); *σ* is stress (MPa); and the peak stress *σ_p_* is used as the term *σ* [30,31].

Equations (2)–(4) are obtained by taking the logarithm of both sides of Equation (1).

(2) $$\ln\dot{\varepsilon }=\ln A_{1}-\frac{Q}{RT}+n_{1}\ln\sigma \;\left(\alpha \sigma <0.8\right)$$

(3) $$\ln\dot{\varepsilon }=\ln A_{2}-\frac{Q}{RT}+\beta \sigma \;(\alpha \sigma >1.2)$$

(4) $$\ln\dot{\varepsilon }=\ln A-\frac{Q}{RT}+n\ln\left[\sinh\left(\alpha \sigma \right)\right]\;\left(\mathrm{for}\;\mathrm{all}\;\sigma \right)$$

**Figure 3:**
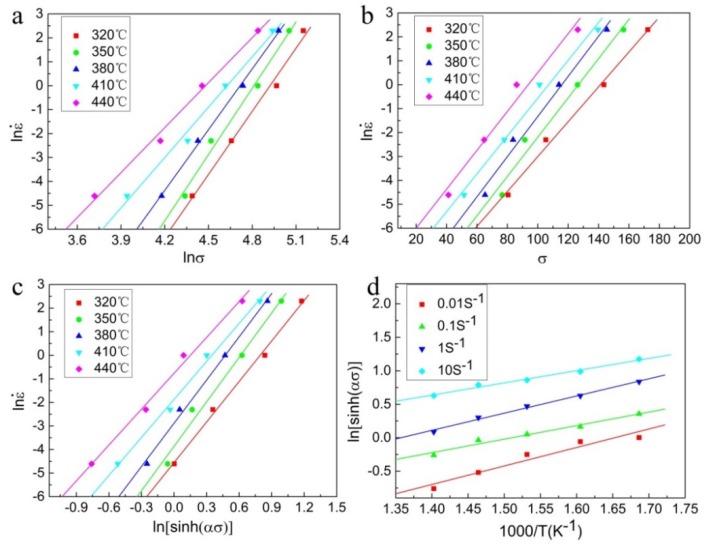
Linear relationship fitting: (**a**) ln$\dot{\varepsilon }–$lnσ, (**b**) ln$\dot{\varepsilon }–$ σ, (**c**) ln$\dot{\varepsilon }–$ ln[sinh(ασ)], (**d**) ln[sinh(ασ)]– 1000/T.

On account of the measured data, the relationship between ln*σ*, *σ*, and $\ln\dot{\varepsilon }$ at different deformation temperatures is plotted in Figure 3a,b. After linear fitting, the values of *n_1_* and *β* can be calculated to be 7.25583 and 0.07963 MPa^−1^, respectively. Therefore, *α* = *β*/*n_1_* = 0.01097 MPa^−1^.

Differentiating Equation (4) gives the following equation:

(5) $$Q=R{\left[\frac{\partial ln\dot{\varepsilon }\;}{\partial ln\left[sinh\left(\alpha \sigma \right)\right]}\right]}_{T}{\left[\frac{\partial ln\left[sinh\left(\alpha \sigma \right)\right]}{\partial \left(1/T\right)}\right]}_{\dot{\varepsilon }}=RnS$$

Thus, the values of *n* and *S* can be obtained from the mean slope of the $\ln\dot{\varepsilon }–$ln[sinh(ασ)] and ln[sinh(ασ)]–1000/T plots, as shown in Figure 3c,d, respectively. The calculated *Q* is 189.07 kJ mol^−1^, which is lower than the value of 318 kJ mol^−1^ for the solution-treated 7085 alloy reported by Liu et al. [32] and similar to the value of 182.07 kJ mol^−1^ for the homogenized 7085 alloy revealed by Park et al. **[33]**. Moreover, the value of *Q* determined in present work is larger as compared to the value of 142 kJ mol^−1^ for the pure aluminum [34]. This difference may be ascribed to the dynamic precipitation, dissolution of precipitates, dislocation pinning effect, heat treatment conditions, and the amount of solute atoms (Zn, Mg, and Cu) in the alloy.

Moreover, the Zener-Hollomon (Z) parameter is as follows [35]:

(6) $$Z=\dot{\varepsilon }exp\left(\frac{Q}{RT}\right)=A{\left[sinh\left(\alpha \sigma \right)\right]}^{n}$$

Then, the logarithm of both sides of Equation (6) is taken. A best linear relationship of lnZ–ln[sinh(ασ)] can be obtained. Hence, the value of A can be easily calculated as 3.79754 × 10^13^.

Thus, the constitutive model of the studied alloy can be expressed as follows:

(7) $$\{\begin{array}{l} \;Z=\dot{\varepsilon }exp\left(\frac{189070}{8.314T}\right)\\ \sigma =\frac{1}{0.01097}\ln\left\{{(\frac{Z}{3.7954\times 10^{13}})}^{\frac{1}{5.71586}}+{\left[{(\frac{Z}{3.7954\times 10^{13}})}^{\frac{2}{5.71586}}+1\right]}^{\frac{1}{2}}\right\} \end{array}$$

### 3.3. Compensation of Strain

It has been reported by many researchers that the deformation behavior and material constants (i.e., *α*, *β*, *n*, ln*A*, and *Q*) among the entire strain range are significantly affected by the strain during hot deformation [25,36,37,38,39,40]. In addition, Figure 2 shows that the true stress is significantly sensitive to the strain, especially at the initial stage of the true stress–strain curves. However, the effect of strain is not taken into account in the above constitutive model. Thus, such model should consider the compensation of strain.

**Figure 4:**
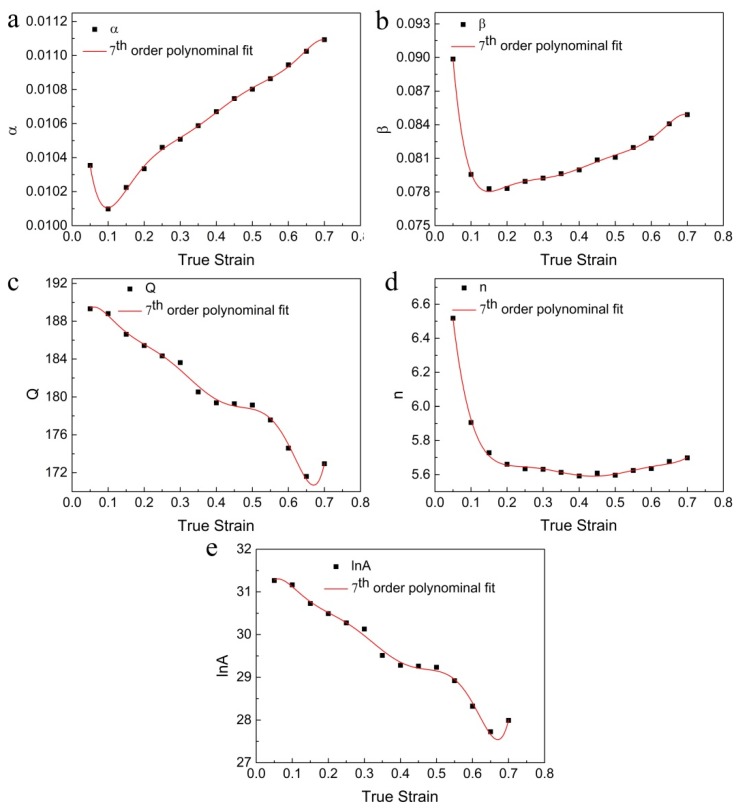
Relationships between true strain and (**a**) *α*, (**b**) *β*, (**c**) *Q*, (**d**) *n*, and (**e**) ln*A* by a seventh-order polynomial fitting.

In previous reports, it has been shown that different order polynomials can be given to well express the effect of strain on these material constants [40,41,42]. So, a seventh-order polynomial is selected for present work, as seen in Equation (8). The values of material constants are computed at strains ranging from 0.05 to 0.7 with an interval 0.05. Then, the coefficients of *α*(*ε*), *β*(*ε*), *n*(*ε*), ln*A*(*ε*), and *Q*(*ε*) can be gained through polynomial fitting. Figure 4 shows the relationships of fitting, and corresponding values are listed in Table 2.

(8) $$\{\begin{array}{l} \alpha \left(\varepsilon \right)=B_{0}+B_{1}\varepsilon +B_{2}\varepsilon^{2}+B_{3}\varepsilon^{3}+B_{4}\varepsilon^{4}+B_{5}\varepsilon^{5}+B_{6}\varepsilon^{6}+B_{7}\varepsilon^{7}\\ \beta \left(\varepsilon \right)=C_{0}+C_{1}\varepsilon +C_{2}\varepsilon^{2}+C_{3}\varepsilon^{3}+C_{4}\varepsilon^{4}+C_{5}\varepsilon^{5}+C_{6}\varepsilon^{6}+C_{7}\varepsilon^{7}\\ Q\left(\varepsilon \right)=D_{0}+D_{1}\varepsilon +D_{2}\varepsilon^{2}+D_{3}\varepsilon^{3}+D_{4}\varepsilon^{4}+D_{5}\varepsilon^{5}+D_{6}\varepsilon^{6}+D_{7}\varepsilon^{7}\\ n\left(\varepsilon \right)=E_{0}+E\varepsilon +E_{2}\varepsilon^{2}+E_{3}\varepsilon^{3}+E_{4}\varepsilon^{4}+E_{5}\varepsilon^{5}+E_{6}\varepsilon^{6}+E_{7}\varepsilon^{7}\\ \ln A\left(\varepsilon \right)=F_{0}+F_{1}\varepsilon +F_{2}\varepsilon^{2}+F_{3}\varepsilon^{3}+F_{4}\varepsilon^{4}+F_{5}\varepsilon^{5}+F_{6}\varepsilon^{6}+F_{7}\varepsilon^{7} \end{array}$$

After the material constants are calculated, the constitutive model considering strain compensation of the studied Al-Zn-Mg-Cu alloy is expressed as Equation (9).

(9) $$\{\begin{array}{l} Z=\dot{\varepsilon }\exp\left(\frac{Q\left(\varepsilon \right)}{8.314T}\right)\\ \sigma =\frac{1}{\alpha \left(\varepsilon \right)}\ln\left\{{(\frac{Z\left(\varepsilon \right)}{A\left(\varepsilon \right)})}^{\frac{1}{n\left(\varepsilon \right)}}+{\left[{(\frac{Z}{A\left(\varepsilon \right)})}^{\frac{2}{n\left(\varepsilon \right)}}+1\right]}^{\frac{1}{2}}\right\} \end{array}$$

### 3.4. Verification of Developed Constitutive Model

**Figure 5:**
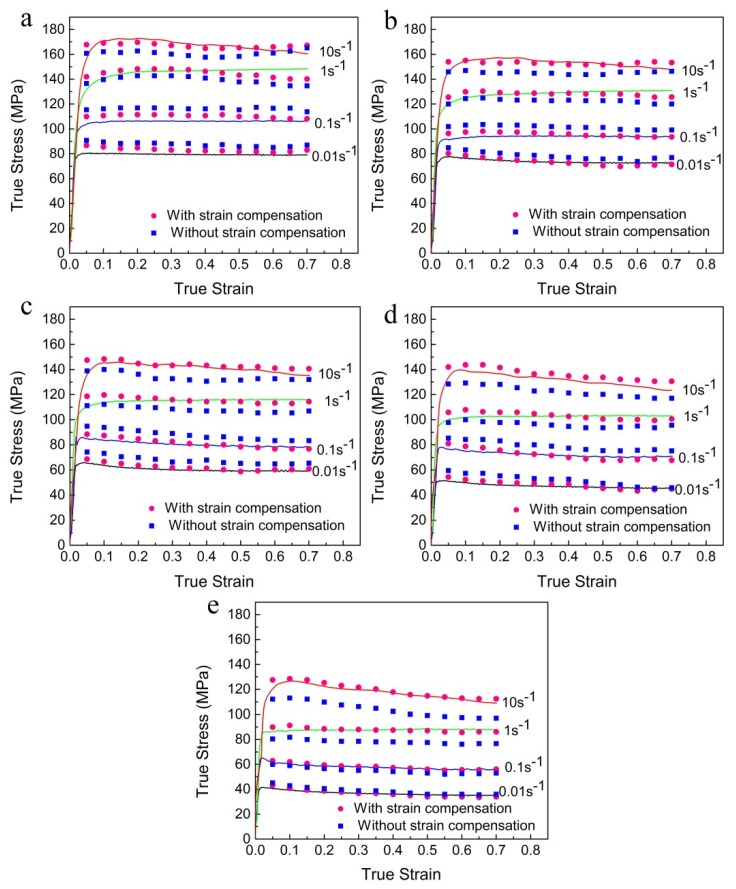
Comparison of the predicted and experimental flow stress curves by two models at deformation temperatures of (**a**) 320 °C, (**b**) 350 °C, (**c**) 380 °C, (**d**) 410 °C, and (**e**) 440 °C.

Figure 5 shows a comparison between the experimental flow stress and predicted values based on the model with and without strain compensation. It can be clearly found that the prediction accuracy of the model with strain compensation is better than that of the model without strain compensation.

Meanwhile, the correlation coefficient (R) and the average absolute relative error (AARE) are selected to quantitatively evaluate the accuracy of two developed constitutive models. They can be represented as [43]:

(10) $$R=\frac{\sum_{i=1}^{N}\left(\sigma_{E}^{i}-\bar{\sigma_{E}}\right)\left(\sigma_{P}^{i}-\bar{\sigma_{P}}\right)}{\sqrt{\sum_{i=1}^{N}{\left(\sigma_{E}^{i}-\bar{\sigma_{E}}\right)}^{2}\sum_{i=1}^{N}{\left(\sigma_{P}^{i}-\bar{\sigma_{P}}\right)}^{2}}}$$

(11) $$AARE\left(\%\right)=\frac{1}{N}\sum_{i=1}^{N}\left|\frac{\sigma_{E}^{i}-\sigma_{P}^{i}}{\sigma_{E}^{i}}\right|\times 100$$

where, $\sigma_{E}^{i}$ is the experimental value and $\sigma_{P}^{i\;}$ is the predicted value. $\bar{\sigma_{E}}$ and $\bar{\sigma_{P}}$ are the average values of $\sigma_{E}^{i}$ and $\sigma_{P\;}^{i}\;$, respectively. N represents the total number of data used in the research.

**Figure 6:**
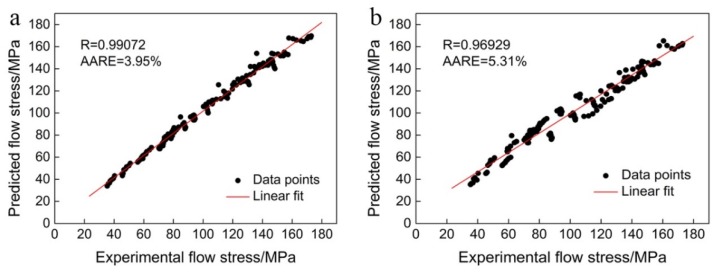
Correlations between the experimental and predicted flow stresses by models (**a**) with and (**b**) without strain compensation.

Figure 6 shows the correlations between the experimental and predicted flow stresses by the model with and without strain compensation. It clear that the values of R and AARE for the model with strain compensation are 0.99072 and 3.95%, respectively, while those of the without strain compensation are 0.96929 and 5.31%, respectively. Obviously, the developed strain compensation constitutive model has higher accuracy than the model without strain compensation. Accordingly, taking the strain compensation into consideration is essential for precisely predicting the flow behavior.

### 3.5. Processing Maps

#### 3.5.1. The Principles of Processing Maps

The processing map is developed based on the DMM proposed by Prasad et al. [44]. According to the principles of DMM, as expressed in Equation (12), the total instantaneous power dissipation (P) is divided into the power dissipated by plastic deformation (G), and the power dissipated through microstructure evolution (J).

(12) $$P=\sigma \dot{\varepsilon }=G+J=\int_{0}^{\dot{\varepsilon }}\sigma d\dot{\varepsilon }+\int_{0}^{\sigma }\dot{\varepsilon }d\sigma$$

The strain rate sensitivity exponent m can be described by Equation (13).

(13) $$m=\frac{\partial \left(\log\sigma \right)}{\partial \left(\log\dot{\varepsilon }\right)}$$

**Figure 7:**
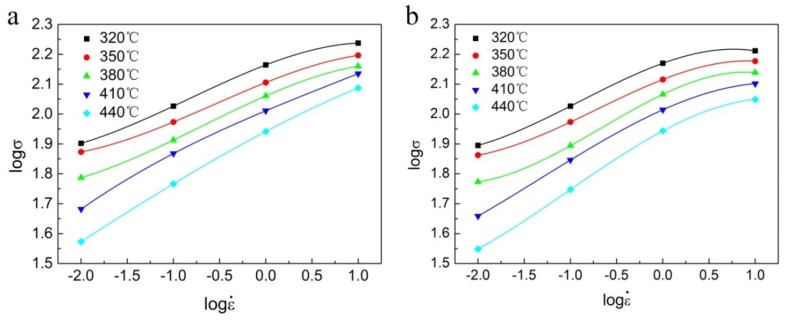
Fitting curves to describe the relationship between logσ and $\log\dot{\varepsilon }$ of Al-Zn-Mg-Cu alloy at strains of (**a**) 0.3 and (**b**) 0.6.

The relationship between true stress and strain rate is given in Equation (14), according to irreversible thermodynamics [44].

(14) $$\log\sigma =a+\mathrm{blog}\dot{\varepsilon }+c{\left(\log\dot{\varepsilon }\right)}^{2}+d{\left(\log\dot{\varepsilon }\right)}^{3}$$

where a, b, c, and d are the polynomial fitting coefficients. The third-order polynomial fitting curves of $\log\sigma –\log\dot{\varepsilon }$ with strains of 0.3 and 0.6 are shown in Figure 7. Corresponding values of the polynomial fitting coefficients are listed in Table 3.

According to Equations (13) and (14), the value of m can be easily derived from Equation (15).

(15) $$m=\frac{\partial \left(\log\sigma \right)}{\partial \left(\log\dot{\varepsilon }\right)}=b+2c\log\;\dot{\varepsilon }+3d{\left(\log\varepsilon \right)}^{2}$$

For an ideal linear dissipater, m = 1 and$\;J_{max}=\sigma \dot{\varepsilon }/2$. While, for a nonlinear dissipater, the power dissipation efficiency *η* can be represented by the following equation:

(16) $$\eta =\frac{J}{J_{max}}=\frac{2m}{m+1}$$

The variation of *η* with deformation temperatures and strain rates at constant strain constitutes the dissipation power map.

A continuum flow instability criterion is defined as:

(17) $$\xi \left(\dot{\varepsilon }\right)=\frac{\partial \log\left(m/m+1\right)}{\partial \log\dot{\varepsilon }}+m\leq 0$$

Plotting $\xi \left(\dot{\varepsilon }\right)$ as a function of deformation temperatures and strain rates at constant strain obtains the flow instability map. When $\xi \left(\dot{\varepsilon }\right)$ is negative, flow instabilities occur during hot deformation.

The processing map is constituted by superimposing the flow instability map over the power dissipation map. In general, the good intrinsic workability should be in the “safe” domains with the peak efficiencies of power dissipation in a processing map [44,45].

#### 3.5.2. Establishment and Analysis of Processing Maps

**Figure 8:**
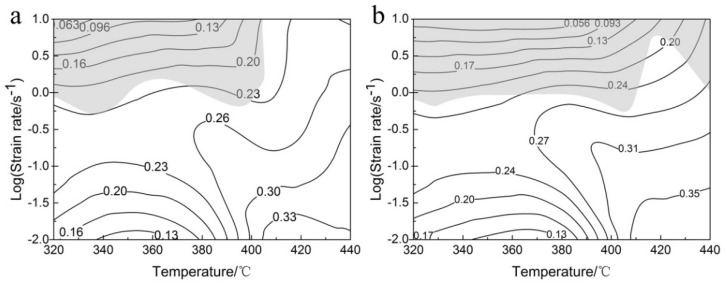
Processing maps of Al-Zn-Mg-Cu alloy at strains of (**a**) 0.3 and (**b**) 0.6.

The processing maps of the samples at strains of 0.3 and 0.6 are shown in Figure 8. In Figure 8, the numbers on the contours are percentage of the power dissipation efficiency, and the shadowed regions represent the flow instability domains, while the white areas represent the “safe” processing domains.

As shown in Figure 8a,b, these processing maps demonstrate a common character that the flow instability domains nearly locate at their upper parts. However, there are differences in the shape of the flow instability domains. The instability domain gradually extends to the high temperature–high strain rate direction with increasing strain. This shows that the processing maps of sample are significantly affected by the strain. Liu et al. also found that the unstable region of 6063 alloy increases with increasing strain [40]. This is due to the concentration of stresses intensified with the increasing strain.

The highest power dissipation efficiency of the samples gradually increases as the strain increases from 0.3 to 0.6, with values of 0.35 and 0.39, respectively. This is ascribed to the microstructural evolution with the increase of strain. It is well known that DRV, DRX, and superplasticity are regarded as the “safe” deformation mechanisms. Previous literature has reported that DRV is identified as main deformation mechanism if the power dissipation efficiency is about 0.30, but DRX is correlated with the higher power dissipation efficiency [46]. Further discussion will be carried out in Section 3.6. When the power dissipation efficiency is greater than 0.6, superplasticity occurs during hot deformation [47]. However, in Figure 8a,b, the peak power dissipation efficiencies of the samples are lower than 0.6, indicating that no superplasticity occurs in these processing maps. Generally, the “safe” domains with the peak efficiencies of power dissipation in a processing map correspond to the ideal workability, where the hot processing parameters are considered as the optimal [44,45]. According to Figure 8b, when the strain is 0.6, the optimal hot processing parameters are deformation temperature of 390–440 °C and strain rate of 0.010–0.316 s^−1^ with a peak efficiency of 0.390.

### 3.6. Microstructural Evolution

**Figure 9:**
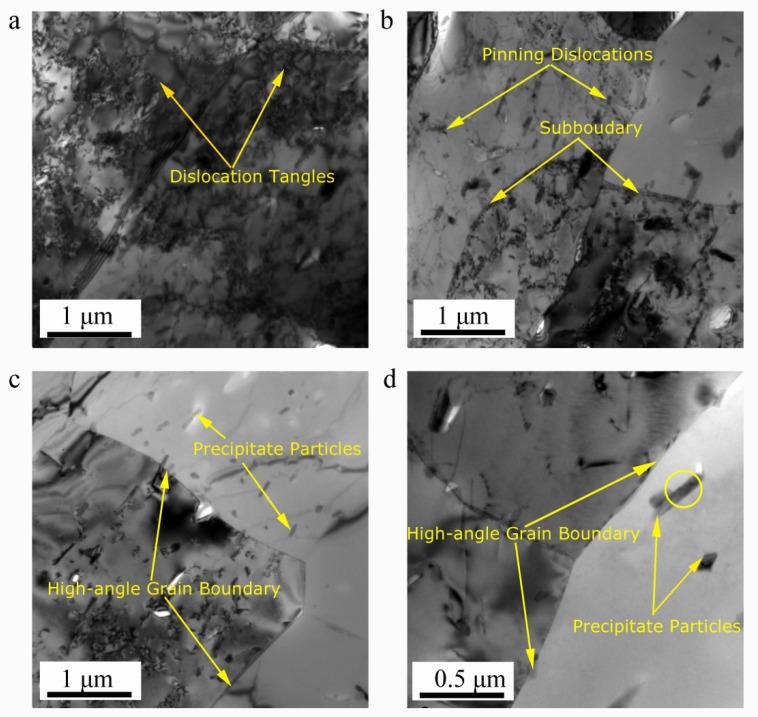
TEM micrographs of Al-Zn-Mg-Cu alloy in various deformation conditions: (**a**) 320 °C/1 s^−1^, (**b**) 380 °C/0.1 s^−1^, (**c**) 410 °C/0.1 s^−1^, (**d**) 440 °C/0.01 s^−1^.

Figure 9 exhibits the TEM results of several deformed samples. It is obvious that microstructural evolution is greatly affected by hot forming parameters. High density dislocation tangles in the deformed grain interior can be easily found when deformed under the condition of 320 °C/1 s^−1^ (Figure 9a). Increasing deformation temperature or decreasing strain rate (380 °C/0.1 s^−1^) (Figure 9b), can provide higher driving force or longer time for dislocation annihilation and rearrangement through climbing and sliding, reducing the dislocation density and forming the dislocation walls within the deformed grain. Subsequently, the dislocation walls convert to subgrain boundaries, segmenting the deformed grains into some subgrain structures; DRV occurs during this period. When the deformation temperature increases to 410 °C (Figure 9c), the sufficient atomic diffusion and dislocation migration lead to the merging of several grains. The low-angle grain boundaries (LAGBs) transform to high-angle grain boundaries (HAGBs) progressively by the absorption of dislocations, indicating that DRV is intensified with the increasing deformation temperature. With a further increase in temperature or a decrease in strain rate (440 °C/0.01 s^−1^) (Figure 9d), the dislocation density decreases, the straight and clear grain boundaries are found, and the recrystallization grains gradually grow through the migration of dislocations and HAGBs. On the basis of these microstructural observation, obviously, the dominant dynamic softening mechanism of the studied alloy is stemmed from DRV and partial DRX during hot deformation.

**Figure 10:**
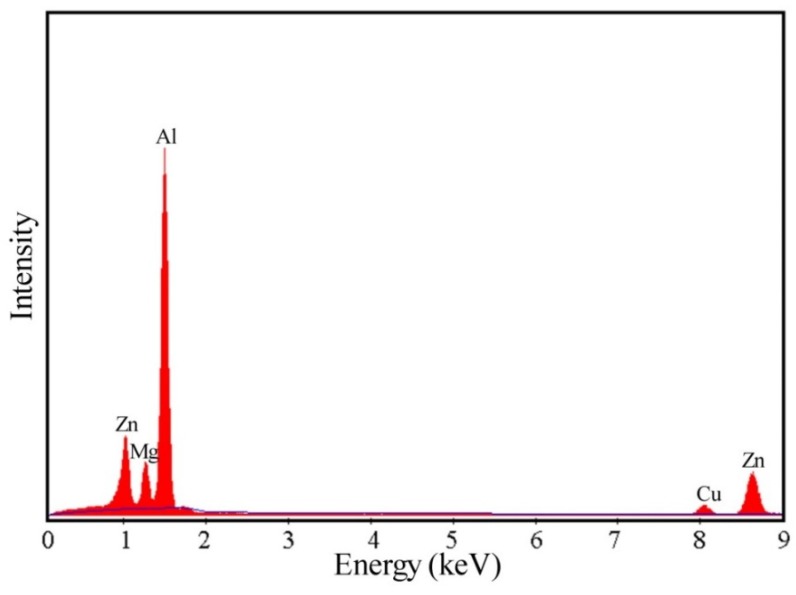
EDS analysis result of the particle marked with circle in Figure 9.

Additionally, another microstructural feature is precipitates. As shown in Figure 9a, many spherical and rod-shaped precipitates are found within subgrains when deformed at 320 °C/1 s^−1^. From Figure 1b and Figure 9, this is a direct result of the sufficient static precipitation prior to hot compression. In the studied alloy, Figure 10 shows the energy dispersive spectrometer (EDS) results of the particles, suggesting the particles contain more Mg, Zn and a small amount of Cu due to the different diffusive ability, with a composition approaching the stoichiometric Mg(Zn, Cu)_2_ phase. This finding is consistent with previous research [20,48,49,50]. These small precipitates induce a strong pinning effect on dislocations and then retard the movement of dislocations, leading to a great flow stress hardening at the initial stage of hot compression. However, in Figure 9, with the increasing deformation temperature or the decreasing strain rate, the precipitate size increases while its number density decreases. According to the Ashby-Orowan criterion, the pinning effect on dislocations will decrease [39]. Consequently, the degree of partial DRX is enhanced, leading to a significant flow stress softening. Moreover, it is noted that the precipitates begin the process of dynamic coarsening and even dissolve into the matrix when deformed at 380 °C/0.1 s^−1^ (Figure 9b), which can also result in a variation of the isoefficiency contours at about 390–440 °C in the processing maps, as shown in Figure 8. As reported by Wu et al. [25], the dissolution of the second phase leads the isoefficiency contours about at 387–427 °C to vary. Similarly, Luo et al.’s work [15] also shows that the isoefficiency contours at 417 °C are greatly affected by the dissolution of T phase.

**Figure 11:**
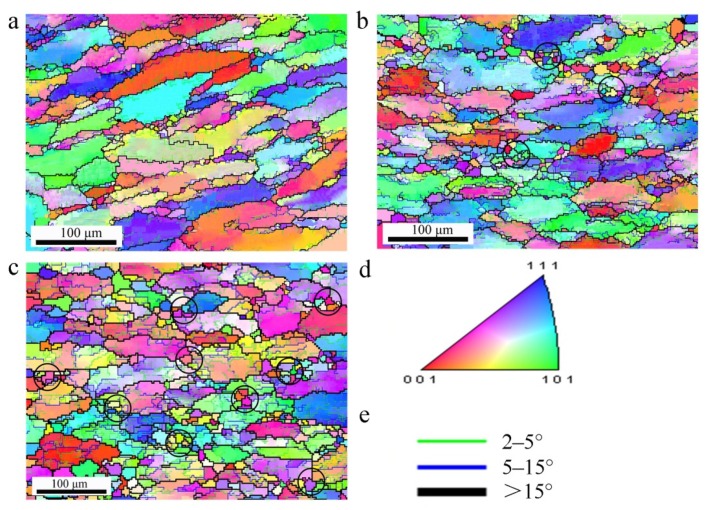
EBSD images of Al-Zn-Mg-Cu alloy in various deformation conditions: (**a**) 380 °C/0.1 s^−1^, (**b**) 410 °C/0.1 s^−1^, (**c**) 440 °C/0.01 s^−1^, (**d**) Inverse pole figure (IPF), (**e**) representation of different color lines used to identify the boundaries with different misorientation angles in (**a**–**c**).

In general, EBSD maps are used to provide more information on microstructural evolution during hot deformation. Figure 11 shows EBSD results of several hot deformed samples. The initial large elongated grains containing equiaxed subgrains are dominant, and the deformed microstructure varies with hot processing parameters. When deformed at 380 °C/0.1 s^-1^ (Figure 11a), many new equiaxed subgrains appear in the form of LAGBs within the elongated grains. In addition, it can be clearly seen that the subgrains prefer to form around the boundaries of deformed grains, owing to the climb and cross-slip of dislocation being more thorough near the elongated grain boundary. When deformed at 410 °C/0.1 s^−1^ (Figure 11b), the LAGBs extend progressively towards the center of the deformed grain, suggesting that DRV is gradually enhanced with the increasing deformation temperature. Meanwhile, some fine DRX grains appear along the elongated original grain boundaries, indicating the transformation from LAGBs to HAGBs under such condition. This phenomenon arises from the fact that a higher deformation temperature accelerates the dislocation annihilation and rearrangement. As a result, the migration of subgrain boundaries and the polygonization are intensified; the degree of DRV is therefore enhanced and DRX occurs. When deformed at 440 °C/0.01 s^−1^ (Figure 11c), DRV further proceeds, the volume fraction of DRX increases, and some DRX grains grow up. The results are attributed to the facts that deformation at higher temperatures promotes the migration of subgrain boundaries, and deformation at lower strain rates provides enough time for the migration of subgrain boundaries. Besides, more precipitates coarsen and even dissolve into the matrix, leading to a weaker pinning effect on subgrain and grain boundaries [51,52,53], which is consistent with the TEM observation in this work. However, in Figure 11, it can also be clearly found that the DRV is still the main softening mechanism despite the occurrence of DRX.

As seen in Figure 11, a color gradient appears between neighboring subgrains in the interior of elongated grains. Additionally, the grain size of the new fine DRX grains is very close to that of the subgrains, and the orientation of DRX grains is different from their adjacent grains. This suggests that the progressive subgrain rotation by the gradual absorption of dislocations leads to a change in their misorientation. The progressive subgrain rotation gives rise to the misorientation rearrangement LAGBs and their gradual transformation to HAGBs, indicating that CDRX is the dominant DRX mechanism during deformation. Similar DRX mechanism has been observed in previous reports [16,17,49]. In general, the CDRX is considered to be an extended DRV, the dominant DRX mechanism for high stacking fault energy metals (e.g., aluminum alloy) is CDRX rather than DDRX owing to the dislocations climb and cross-slip that are prone to occur in such alloys. Moreover, the new fine DRX grains marked with black circles in Figure 11b,c form at triple junctions and original elongated grain boundaries, suggesting that DRX occurs preferentially at such sites.

**Figure 12:**
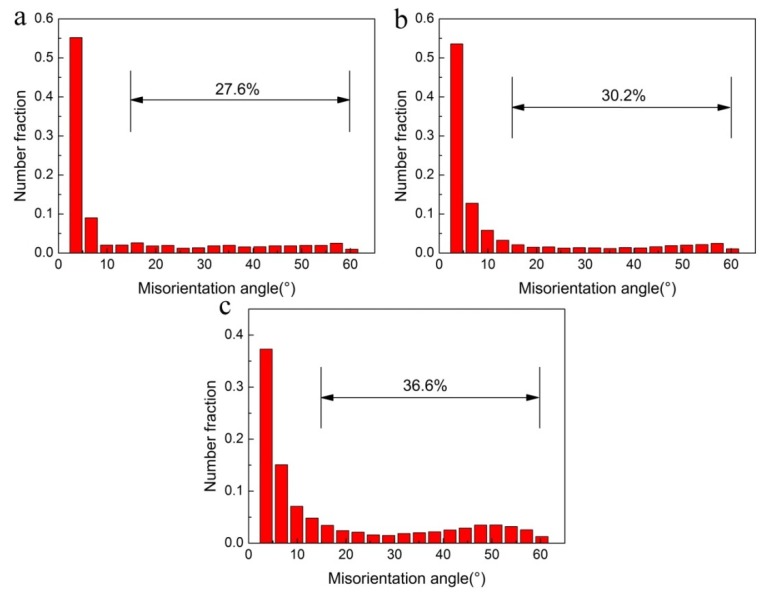
Misorientation angle distributions of Al-Zn-Mg-Cu alloy under various deformation conditions: (**a**) 380 °C/0.1 s^−1^, (**b**) 410 °C/0.1 s^−1^, (**c**) 440 °C/0.01 s^−1^.

Figure 12 presents the misorientation angle distributions of the grain boundaries under different deformation conditions. According to Figure 12, the average misorientation angles of the deformation conditions of 380 °C/0.1 s^−1^, 410 °C/0.1 s^−1^, and 440 °C/0.01 s^−1^ are 12.3°, 13.6°, and 17.7°, respectively. Meanwhile, the fractions of HAGBs can be evaluated as 27.6%, 30.2%, and 36.6%, respectively. It is evident that the majorities (~70%) of the boundaries are LAGBs for all conditions, which indicates that DRV and slight DRX have taken place during the process of samples. The medium angle boundaries (MAGBs, 5–15°) usually reflect the occurrence of CDRX during the hot deformation [53,54,55]. According to the above result, this shows that some LAGBs gradually converted into MAGBs, which, in turn, evolved into HAGBs with the increasing deformation temperature or the decreasing strain rate. Therefore, this evolution can also be regarded as a process of CDRX, which is consistent with the analysis in Figure 11. Similar behavior is also reported in other alloys, such as 6099 alloy [8].

**Figure 13:**
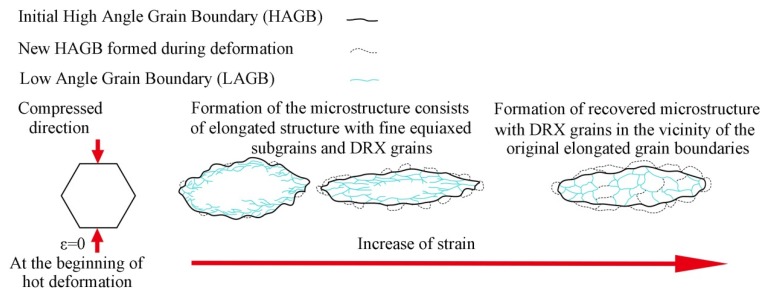
Schematic illustration of the microstructural evolution of Al-Zn-Mg-Cu alloy during hot deformation.

Based on the above analysis, the microstructural evolution of samples during hot compression is proposed in Figure 13. With the increasing strain, the equiaxed grains are obviously elongated perpendicularly to the compressed direction owing to hot deformation, and LAGBs transform to HAGBs, leading to the formation of new fine DRX grains. The new small grains with HAGBs are formed preferentially near the original elongated grain boundaries. The transformation of LAGBs to HAGBs is an indication of the occurrence of CDRX in studied alloy. In summary, both DRV and DRX occur during hot deformation. DRV is identified as main dynamic softening mechanism. The optimal hot processing parameters deduced from the stable domain of the processing map are reliable.

## 4. Conclusions

In present work, the deformation behaviors considering strain effects and recrystallization mechanism of an Al-Zn-Mg-Cu alloy are investigated. The main results are summarized as follows:

- The Mg(Zn, Cu)_2_ particles as a result of the sufficient static precipitation prior to hot compression have an influence on the flow behavior by effectively pinning the dislocation during hot compression. Both dynamic coarsening and even dissolution of Mg(Zn, Cu)_2_ particles can also cause the flow stress softening.
- The R and AARE values of the developed constitutive model compensated with strain are 0.99072 and 3.95%, respectively, suggesting a relatively high precision of the developed constitutive model. The constitutive model of the studied alloy can be expressed as:
  (18) $$\{\begin{array}{l} Z=\dot{\varepsilon }\exp\left(\frac{Q\left(\varepsilon \right)}{8.314T}\right)\\ \sigma =\frac{1}{\alpha \left(\varepsilon \right)}\ln\left\{{(\frac{Z\left(\varepsilon \right)}{A\left(\varepsilon \right)})}^{\frac{1}{n\left(\varepsilon \right)}}+{\left[{(\frac{Z}{A\left(\varepsilon \right)})}^{\frac{2}{n\left(\varepsilon \right)}}+1\right]}^{\frac{1}{2}}\right\} \end{array}$$
- Both DRV and DRX occur, and DRV is identified as the main dynamic softening mechanism. The recrystallization mechanism is CDRX. CDRX prefers to occur at triple junctions and original elongated grain boundaries.
- Based on the processing map and microstructural observation, the optimal hot processing parameters at a strain of 0.6 are in the deformation temperature range of 390–440 °C and the strain rate range of 0.010–0.316 s^−1^ with a peak efficiency of 0.390.

## Figures and Tables

**Table 1:** Chemical compositions of the studied Al-Zn-Mg-Cu alloy (in wt %).

Composition	Zn	Mg	Cu	Mn	Cr	Ti	Si	Fe	Al
Content	7.00	2.37	1.66	0.40	0.15	0.11	0.026	0.046	Bal.

**Table 2:** Coefficients of polynomial fit for *α*, *β*, *Q*, *n*, and ln*A* in Equation (8) for Al-Zn-Mg-Cu alloy.

α	β	Q	n	lnA
B_0_ = 0.01173	C_0_ = 0.12283	D_0_ = 182.61081	E_0_ = 8.09318	F_0_ = 29.92634
B_1_ = −0.04759	C_1_ = −1.04332	D_1_ = 299.84774	E_1_ = −47.05679	F_1_ = 59.96955
B_2_ = 0.51783	C_2_ = 9.73699	D_2_ = −4614.03387	E_2_ = 386.38381	F_2_ = −922.80677
B_3_ = −2.71861	C_3_ = −47.39541	D_3_ = 31,736.66147	E_3_ = −1725.87388	F_3_ = 6347.33229
B_4_ = 7.90719	C_4_ = 131.40288	D_4_ = −117,379.43127	E_4_ = 4480.68971	F_4_ = −23,475.88625
B_5_ = −12.95283	C_5_ = −208.49173	D_5_ = 236,927.83041	E_5_ = −6756.57195	F_5_ = 47,385.56608
B_6_ = 11.18036	C_6_ = 176.18315	D_6_ = −244,644.91171	E_6_ = 5491.34758	F_6_ = −48,928.98234
B_7_ = −3.95213	C_7_ = −61.42370	D_7_ = 100,787.54684	E_7_ = −1859.00011	F_7_ = 20,157.50937

**Table 3:** Polynomial fitting coefficients in Equation (14) for Al-Zn-Mg-Cu alloy.

Strain	Temperature (°C)	Coefficient			
		a	b	c	d
0.3	320	2.16450	0.11845	−0.03227	−0.01296
	350	2.10561	0.12358	−0.02061	−0.01216
	380	2.06092	0.13526	−0.02421	−0.01172
	410	2.01119	0.12968	−0.00947	−0.00399
	440	1.94186	0.16220	−0.01477	−0.00183
0.6	320	2.17009	0.11138	−0.05082	−0.01888
	350	2.11574	0.12059	−0.04041	−0.01870
	380	2.06603	0.14718	−0.04948	−0.02495
	410	2.01444	0.13799	−0.04029	−0.01019
	440	1.94369	0.16558	−0.04555	−0.01477
